# Teaching to make stone tools: new experimental evidence supporting a technological hypothesis for the origins of language

**DOI:** 10.1038/s41598-017-14322-y

**Published:** 2017-10-31

**Authors:** D. Lombao, M. Guardiola, M. Mosquera

**Affiliations:** 1 IPHES, Institut Català de Paleoecologia Humana i Evoluciò Social, Zona educacional 4 (Edif. W3), Campus Sescelades URV, 43007 Tarragona, Spain; 2 Universitat Rovira i Virgili (URV), Campus Catalunya, Avinguda de Catalunya, 35, 43002 Tarragona, Spain; 3 Laboratory Archéologie et Peuplement de l’Afrique, Department of Genetics and Evolution, Anthropology Unit, University of Geneva, Geneva, Switzerland

## Abstract

The relationship between lithic technology, learning and language is a topic of growing interest in human evolution studies, and has therefore been the subject of numerous scientific papers in recent years. To evaluate the role of language in the social transmission of lithic technology, we designed and developed an experimental protocol through which we compared the acquisition of knapping skills in thirty non-experts in the early stages of learning, by means of three mechanisms of social transmission: imitation-emulation, gestural communication, and verbal communication. All the apprentice knappers carried out the experimental task with blanks that were equal in shape and size, and were requested to replicate what the expert knapper was doing: the alternating method, a sufficiently simple, but systematic technique for detaching flakes from a core. We analysed each participant’s actions, including those of the master knapper, the final products (flakes and cores), and the knapping sequences, by analysing the refits. Our results show that the apprentices improved their knapping skills in teaching conditions -both gestural and verbal communication-, and specially through the latter. In conclusion, our study supports the hypothesis of co-evolution between lithic technology and social learning, which could have favoured the emergence of verbal language.

## Introduction

Complex lithic technological capacity and language compete with each other to be the insignia of human intelligence, due to their cognitive implications. While stone tools have remained more or less unchanged in the archaeological record and act as a window into the behaviour of pre-modern hominins^1^, language does not fossilise. This means indirect approaches are necessary to approximate this capacity in extinct hominins^2–4^. This hinders the study of the relationship between lithic technology and language in evolutionary terms, and this currently remains controversial^5,6^. Several experimental studies in cognitive neuroscience have focused on Broca’s area in the frontal lobe which is involved in language production^7–10^, and manual praxis^11–13^, such as those involved in tool production^10,14–16^.

Some experimental works focusing on Broca’s area have looked at the overlap between language and the production of Lower Palaeolithic tools^17^, both for Oldowan^18,19^ and Acheulian industries^20,21^ with opposite results. Furthermore, Stout and colleagues have also explored the brain processes involved in the acquisition of knowledge related to the knapping methods associated with these technologies^22^. In addition, language and the production of Acheulian tools have been shown to cause the same lateralization of blood flow in the brain^23^.

This body of study comprises evidence supporting the technological hypothesis of the origin of language, and particularly the technological pedagogy hypothesis^6^, which contends that intentional pedagogical demonstration may have spurred the evolution of the verbal communication^24^. In fact, some authors have proposed that social learning and pedagogy would have been key factors in the evolution of hominin brains^25–27^.

Furthermore, some ethnographic studies have reinforced the relationship between lithic technology and language, emphasising the social character of knapping in current human communities^28–33^. In these groups, verbal interaction is a key component of the knapping learning process, especially for transmitting complex technological concepts^31^.

However, only three experimental studies have directly analysed the role of language in the acquisition of knapping knowledge. Ohnuma *et al*.^34^ compared the effectiveness of verbal and gestural communication when transmitting the Levallois method described as *“the classic type”* by Bordes^35^ or “*the method lineale”* by Boëda^36,37^. Recently, Putt *et al*.^38^ studied the differences between verbal language and gestural communication during experimental bifacial stone tool manufacture (handaxes). Finally, Morgan *et al*.^39^ analysed the efficacy of social transmission in replicating Oldowan tools by living humans, using five learning conditions: reverse engineering, imitation-emulation, basic teaching, gestural communication, and verbal communication.

Even with this scarcity of research, these studies show *a priori* opposing results. Ohnuma *et al*.^34^ and Putt *et al*.^38^ found that verbal language did not represent an advantage over gestural communication. However, the results of Morgan *et al*. indicate that gestural communication, and especially verbal communication, are more effective than non-teaching conditions (reverse engineering or imitation-emulation)^39^. This discrepancy may indicate that the participants in the verbal communication group in the Putt *et al*. study^38^ may have correctly understood verbally transmitted strategies, but their lack of practical experience limited their technical ability to carry them out correctly^39,40^.

Furthermore, these studies fail in two aspects. Firstly, none of these experiments considered the need to provide all of the volunteers with nodules of identical shape, texture and size, although it is well known that the initial morphology of a blank strongly influences the final products^41,42^. Secondly, a complete reduction process, such as Levallois or bifacial knapping, actually includes various knapping methods and different ways of managing faces and sides. All of this leads to a huge diversity of options, making the *chaînes opératoires* very difficult to compare. Consequently, it is difficult to quantify the efficacy of the different learning conditions for social transmission in such studies, as a particular product can be obtained in a variety of different ways.

For these reasons, our experiment was designed to evaluate the effectiveness of imitation-emulation, gestural communication and verbal language as conditions of social transmission for learning a specific knapping method, the alternating method, with all participants working with identical blanks. In addition, we focused on evaluating the differences between different learning groups in both the acquisition of technical skills and the understanding of the knapping method. This idea of two independent but related areas of skill in lithic knapping was first introduced by Pelegrin^43^, who defined them as *connaisance* (knowledge) and *savoir-faire* (know-how). The term *connaisance* refers to the knowledge and understanding of concepts related to the knapping activity of a particular technology. This is sometimes described as cognitive knowledge^44^. Meanwhile, the term *savoir-faire* designates the physical skills required for a technology, described as practical know-how.

Our experimental programme involved 30 participants, ranging in age between 20 and 42, randomly divided into three groups of ten individuals each, according to the learning conditions to be imposed during the experiment: 10 volunteers for the imitation-emulation condition, 10 volunteers for the gestural-communication condition and 10 volunteers for the verbal communication condition. None of the participants had prior experience of lithic knapping, and all had the same objective: to reproduce what the expert knapper was doing, i.e., the alternating method (Supplementary Material Table S1).

By the “alternating method”, we do not mean the general discoidal knapping, but the bifacial reduction process in which flakes are removed from the blank turning both faces after each removal^45^, and using the negative of the previous removal as percussion platform for the following detachment. This method is useful for reducing blanks with tabular morphologies, which have very abrupt or straight angles^41,46–49^. In the archaeological record, the earliest evidence of the alternating method identified so far comes from Developed Oldowan sites such as Kanjera South (Kenya), dated as being about 2 My^50^, where short sequences using the alternating method were identified. In addition, the application of this method has been recorded in some early Acheulian sites, such as Gadeb (Ethiopia), dated between 1.4 and 0.7My^51^, and Gesher Benot Ya’aqov (Israel), dated to 0.8 My^52^.

## Results

### Technical capabilities

**Figure 1:**
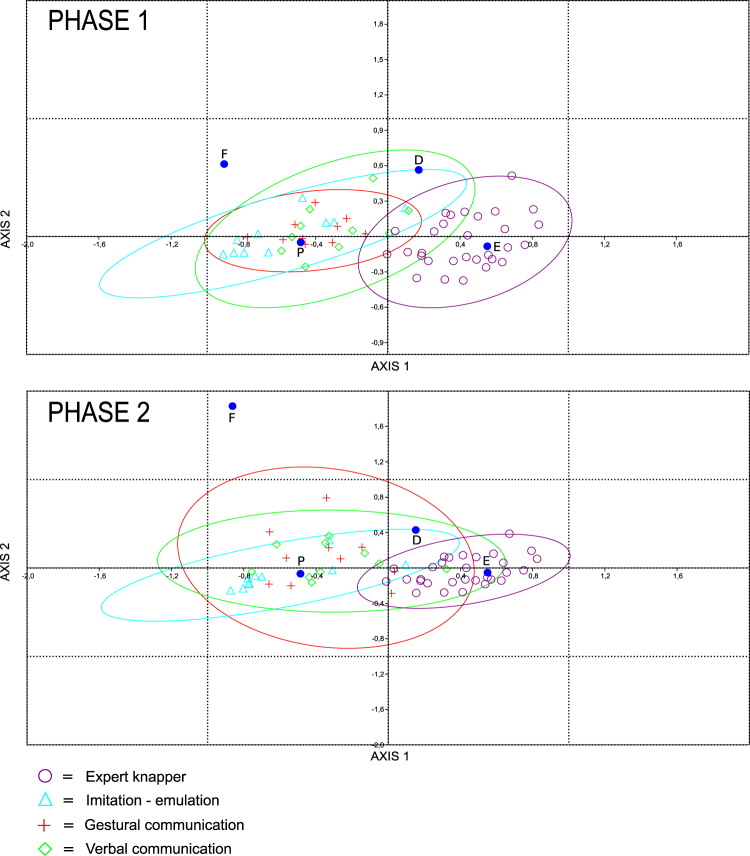
Correspondence Analyses of Technical capacities. P: percussion without extraction; D: percussion resulting in knapping error; E: percussion with flake removal, and; F: failure. Up: Phase 1. Down: Phase 2.

During the experiment, the apprentices in the imitation-emulation group performed considerably higher number of percussion actions (*n* = 3,041), than those in the gestural (*n* = 1,744) or verbal learning groups (*n* = 1,369) (Table 1). The Correspondence Analysis shows the distribution for the apprentices and the expert knapper based on the number and type of actions performed at each phase of the experiment (Fig. 1). The first two variables (percussion without removal and percussion with successful extraction), which explain the distribution of the three groups of apprentices and the expert knapper, represent 91.15% of the total inertia (that is the correlation coefficient between species scores and sample scores) in Phase 1 (when the learners are knapping with the expert knapper), and 89.82% of the total inertia in Phase 2 (when the apprentices are knapping alone).

**Table 1:** Number and types of percussions, for the expert knapper (EK) and apprentices (App.) for each condition of learning (Cond.).

**Exp**.	**Cond**.	**Phase**	**Percussion without extraction (P)**			**Percussion resulting in knapping error (D)**			**Percussion with flake removal (E)**			**Failure (F)**			**Total**		
			**T**	**M**	**SD**	**T**	**M**	**SD**	**T**	**M**	**SD**	**T**	**M**	**SD**	**T**	**M**	**SD**
EK	—	—	315	10.5	6.05	99	3.3	2.18	610	20.3	3.93	0	0	0	1,024	34.1	8.25
App.	I-E	1	946	94.6	59.92	74	7.4	2.5	125	12.5	8.73	10	1	1.49	1,155	115.5	60.59
App.	I-E	2	1,592	159.2	101.7	107	10.7	4.92	179	17.9	8.71	8	0.8	1.22	1,886	188.6	100
TotaI	I-E	—	2,538	126.9	87.75	181	9	4.16	304	15.2	8.93	18	0.9	1.33	3,041	152	88.83
App.	G	1	584	58.4	32.57	72	7.2	2.2	156	15.6	3.33	12	1.2	1.87	824	82.4	32.76
App.	G	2	632	63.2	62.33	102	10.2	11.79	168	16.8	7.39	18	1.8	2.65	920	92	80.27
Total	G	—	1,216	60.8	48.46	174	8.7	8.39	324	16.2	5.61	30	1.5	2.25	1,744	87.2	59.87
App.	V	1	457	45.7	4.59	68	6.8	3.88	167	16.7	2.86	8	0.8	1.54	700	70	25.38
App.	V	2	455	45.5	34.81	65	6.5	4.4	139	13.9	4.43	10	1	1.33	669	66.9	38.82
Total	V	—	912	45.6	28.77	133	6.65	4.04	306	15.3	3.9	18	0.9	1.41	1,369	68.45	31.96
Total	App.	—	4,666	77.7	69.06	488	8.13	5.89	934	15.56	6.4	66	1.1	1.7	6,154	102.5	73

I-E: imitation-emulation group; G: gestural communication group; V: verbal communication group. T: total; M: mean; SD: standard deviation.

The pattern in both phases is the same: the expert knapper is closely related to successful extractions, while the imitation-emulation group is characterized by percussions without extraction. Both the gestural and verbal communication groups are in an intermediate range, with no clear differences between them (see Fig. 1). However, the differences between these groups and the imitation-emulation group are statistically significant in the number of percussions without extraction, both in the mean (Kruskal-Wallis test (p) <0.05) and distribution of values (Kolmogorov-Smirnov (p) <0.05) (Supplementary Material Table S4).

**Figure 2:**
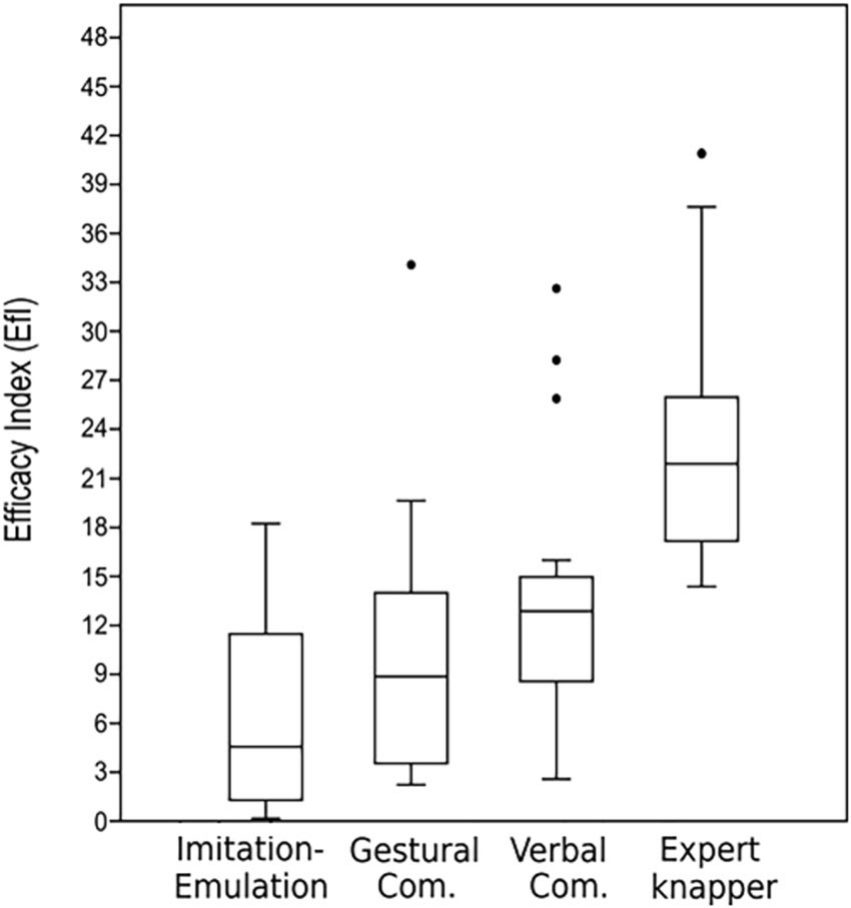
Boxplot of Efficacy Index (EfI) for both each learning condition and the expert knapper.

When the number of actions performed in each knapping session was related to the mass extracted from the corresponding core (Efficacy Index - EfI), the expert knapper displayed a significantly higher mean than the apprentices (EfI EK = 23. 04), indicating his greater effectiveness in reducing the core (Fig. 2). In the three learning groups, on average, the participants in the verbal communication group achieved a higher EfI (EfI V = 13.99) than the members of the gestural communication group (EfI G = 10.43), although these differences are not statistically significant (K-W G-V (p) = 0.07205). The volunteers in the imitation-emulation group needed to perform more percussions than any of the other participants to reduce the core (EfI I-E = 5.98). The results of the K-W test indicate that the differences between the verbal and gestural communication groups and the imitation-emulation group are statistically significant (K-W I-E-G (p) = 0.0239; K-W I-E-V (p) = 0.0003382). (Supplementary Material Tables S5,S6).

### Sequences

The study of flake-core refitting and the subsequent reconstruction of the knapping sequences verified that the verbal communication group produced the highest number of alternating flakes (229), followed by the gestural group (112). In contrast, the imitation-emulation group produced only 13 alternating flakes (Table 2). Striking differences arise when differentiating the results according to the phase of the experiment in which they were produced: although the general tendency in all groups was to produce fewer alternating flakes in Phase 2 than in Phase 1, there were differences in the intensity of these decreases.

**Table 2:** Number of alternating flakes, non-alternating flakes and total flakes, for the expert knapper (EK) and apprentices (App.) for each condition of learning (Cond.).

Exp.	Cond.	Phase	Alternating flakes			No alternating flakes			Total flakes		
			**T**	**M**	**SD**	**T**	**M**	**SD**	**T**	**M**	**SD**
EK	—	—	584	19.4	3.98	45	1.5	2.08	629	20.9	3.55
App.	I-E	1	9	0.9	2.84	153	15.3	7.97	162	16.2	8.31
App.	I-E	2	4	0.4	1.26	191	19.1	5.82	195	19.5	6.24
Total	I-E	—	13	0.65	2.15	344	17.2	7.06	357	17.85	7.35
App.	G	1	103	10.3	4.66	72	7.2	5.37	175	17.5	2.36
App.	G	2	9	0.9	1.91	163	16.3	6.68	172	17.2	5.97
Total	G	—	112	5.6	5.94	235	11.75	7.52	347	17.35	4.42
App.	V	1	145	14.5	3.71	37	3.7	4.08	182	18.2	2.82
App.	V	2	84	8.4	5.91	75	7.5	5.75	159	15.9	3.38
Total	V	—	229	11.45	5.73	112	5.6	5.23	341	17.05	3.25
Total App.	—	—	354	5.9	6.57	691	11.51	8.12	1045	17.41	5.21

I-E: imitation-emulation group; G: gestural communication group; V: verbal communication group. T: total; M: mean; SD: standard deviation.

In the imitation-emulation learning group, the difference in alternating flake production between Phase 1 and Phase 2 was not so pronounced, although it is important to consider that their overall percentage of alternating flakes was very low, dropping from 5.56% in Phase 1 to 2.05% in Phase 2 (a relative decrease of 63.1% in Phase 2 compared to Phase 1). In the verbal communication group, the decrease is more pronounced in absolute terms, as alternating flakes dropped from 79.56% in Phase 1 to 53.13% in Phase 2 (representing a relative decrease of 33.7% in Phase 2 compared to Phase 1). Finally, the greatest decrease occurred in the gestural communication group, falling from 58.86% of alternating flakes in Phase 1 to just 5.23% in Phase 2 (representing a relative drop of 91.1%). Anyway, all percentages are lower than that achieved by the expert knapper (92,85% of alternating flakes).

**Figure 3:**
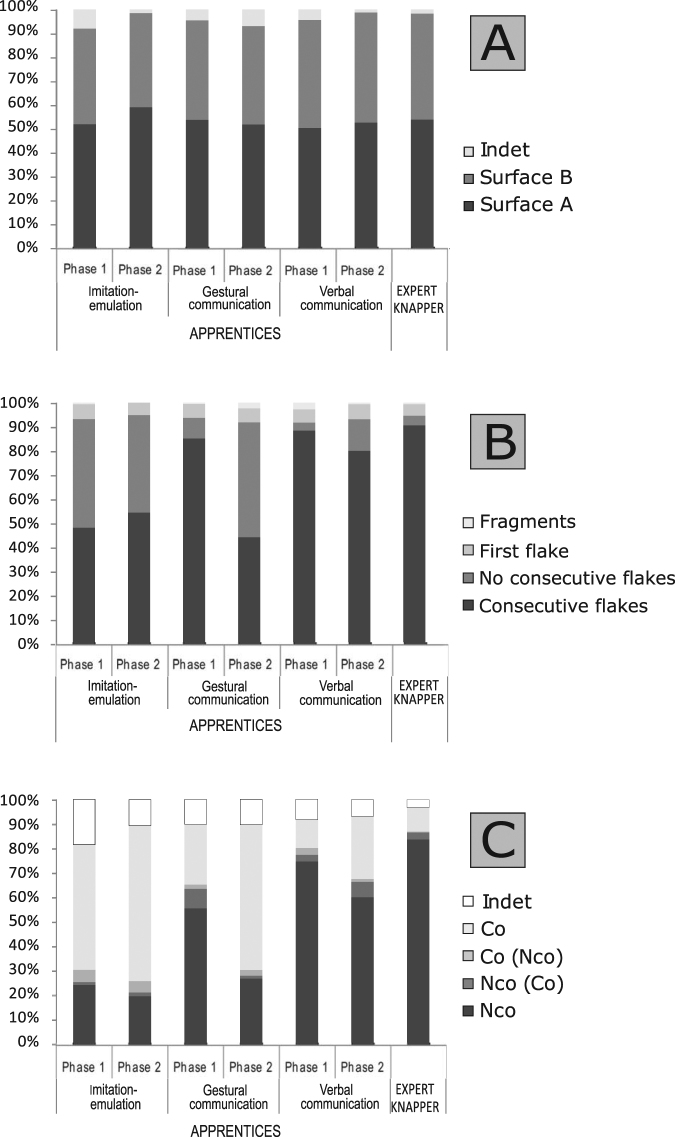
(**A**) Proportion of flakes from each core surface. (**B**) Proportion of consecutive flakes for each learning condition. (**C**) Proportion of types of cortical striking platform. Nco: Completely non-cortical; Nco (Co); non-cortical dominant; Co (Nco): cortical dominant; Co: completely cortical; and Indet.: indeterminate.

In order to investigate the possible causes of these differences we independently analysed the three criteria applied in this study to determine whether the alternating method was being performed. In *alternating the faces of the core*, all three groups showed the same pattern of equilibrium among faces, with a slight predominance of flakes from percussions on face A, a very similar pattern to the expert knapper (Fig. 3A). In *core rotation*, that is, the continuity of extractions around the perimeter of the core, the verbal communication group obtained a higher total number of consecutive flakes (288), while the imitation-emulation group and the gestural communication group performed a total of 184 and 249 consecutive removals, respectively. In the gestural group, there was a decrease from 85.14% of consecutive flakes in Phase 1 to 52.91% in Phase 2, the latter being very similar to the imitation-emulation group (48.15% in Phase 1 and 54.36% in Phase 2). The verbal group showed a slight decrease from 88.40% in Phase 1 to 80% in Phase 2, getting in both phases a percentage very similar to the expert (90% of consecutive flakes) (Fig. 3B).

However, the most significant differences were found in the c*ortical area of the flake percussion platforms*, which indicate if the percussion was made on a previous scar. In the case of the expert knapper the flakes are dominated by non-cortical platforms (83.62%), with few completely cortical platforms (9,86%). In the imitation-emulation group, completely cortical platforms predominated in both Phase 1 (Co: 52.23%) and Phase 2 (Co: 63.59%), whereas in the verbal communication group completely non-cortical platforms predominated in both phases (Phase 1 Nco: 74.59%; Phase 2 Nco: 60%). Furthermore, non-cortical platforms predominated in Phase 1 in the gestural group (Nco: 55.43%), while in Phase 2 completely cortical platforms predominated (Co: 59.3%) (Fig. 3c). Therefore, the “*core rotation*” and, particularly, “*cortical area of the flake percussion platforms*” variables best reflect the decreasing number of alternating flakes during the reduction process. (Supplementary Material Tables S8,S9 and S10).

### Products

**Figure 4:**
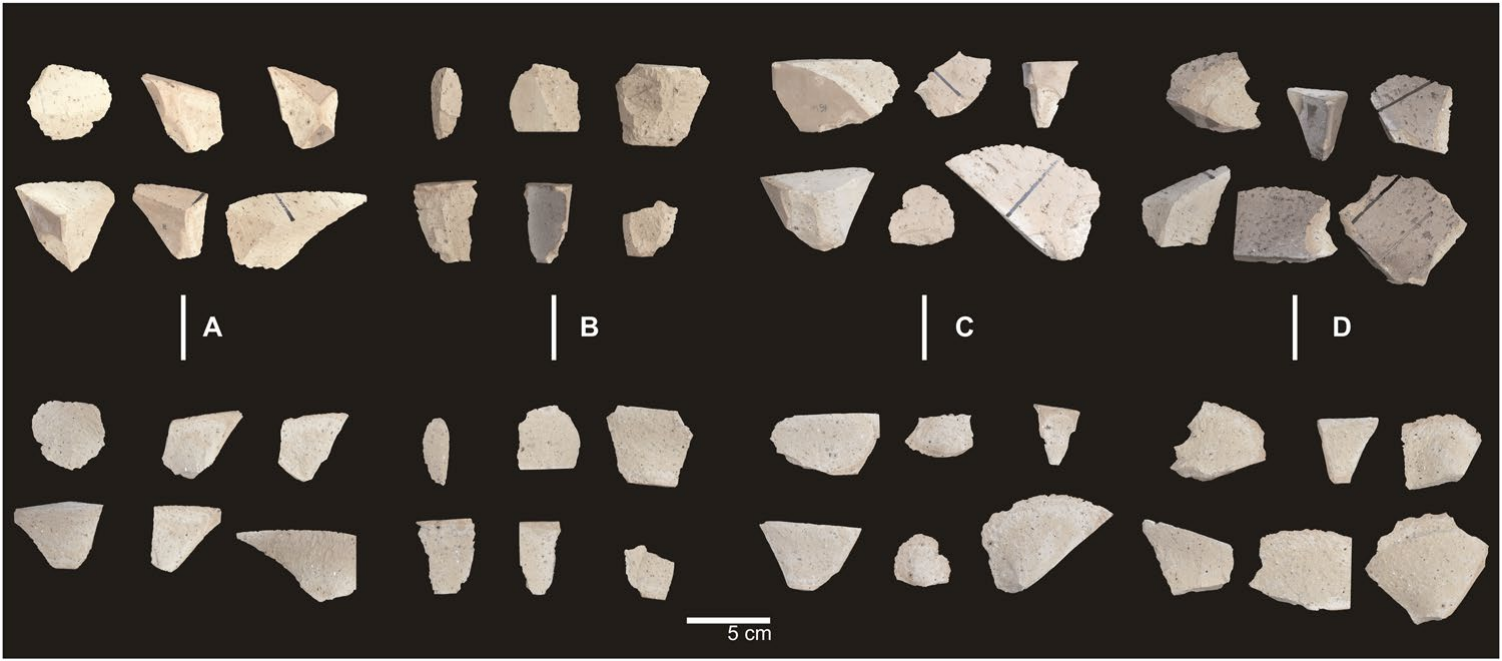
Flakes from: (**A**) expert knapper, (**B**) imitation-emulation group, (**C**) gestural communication group, and (**D**) verbal communication group.

**Figure 5:**
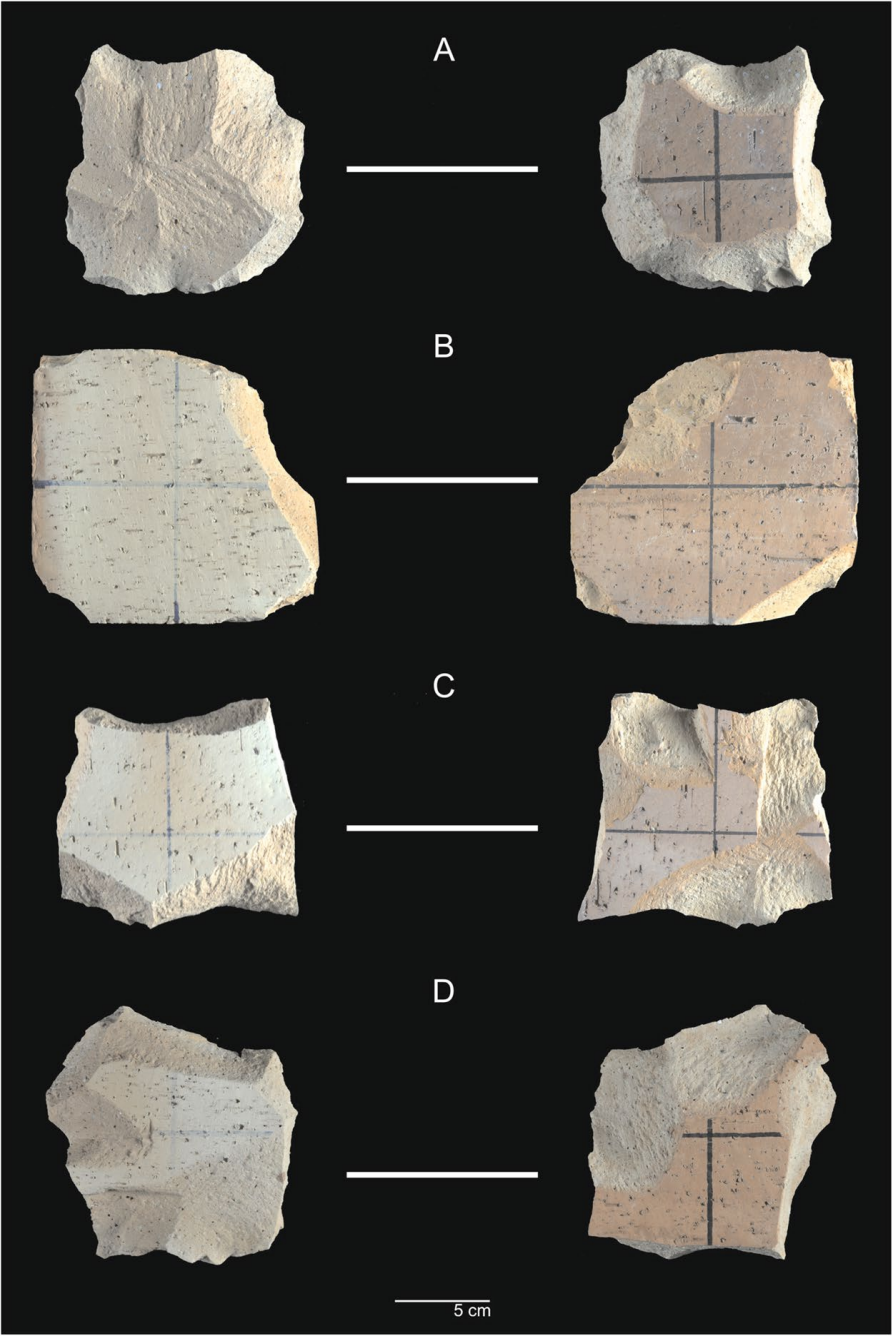
Cores from (**A**) expert knapper, (**B**) imitation-emulation group, (**C**) gestural communication group, and (**D**) verbal communication group.

A total of 1674 flakes and 90 cores were produced during the experiment. The skilled knapper generated 629 flakes and 30 cores; the imitation-emulation apprentices produced 357 flakes; the gestural communication apprentices generated 347 flakes; and the verbal communication apprentices obtained 341flakes (Fig. 4). Each learning group produced 20 cores (Fig. 5).

We compared the attributes of the flakes obtained by the different learning groups between each other, and also to those of the flakes produced by the expert knapper. The products of the verbal communication group were most like those of the skilled knapper. In fact, there were no statistically significant differences (K-W > 0.05; K-S > 0.05) in width, thickness, carenate index (a sort of ratio that allows to differentiate between thick and thin pieces in relative terms, since it correlates the smaller dimension of length and width divided by the thickness), and platform thickness. In contrast, the products of the imitation-emulation group were only similar to those of the skilled knapper in platform width and elongation index (a sort of ratio that allows to differentiate between long and short pieces, since it correlates the technical length divided by the technical width). Similarities between the flakes produced by the gestural communication group and those made by the expert knapper were only documented in the platform thickness and elongation index (Supplementary Material Table S11).

The most interesting difference among the learning groups is that while the verbal and gestural communication participants show thicker striking platforms, similar to those of the skilled knapper (K-W G-EK (p) = 0.9257; K-W V-EK (p) = 0.3566), the imitation-emulation group produced thinner percussion platforms, striking closer to the edge of the core.

Besides the gestural and verbal communication groups show similar striking platforms, there are statistical significant differences between the verbal communication group and the imitation-emulation and gestural communication groups, either in the thickness of the flakes (K-W IE-V (p) = 0.003855; K-W G-V (p) = 0.005541), and the carenate index (K-W IE-V (p) = 0.000000433; K-W G-V (p) = 0.001969). However, these differences are not statistically significant between the imitation-emulation group and the gestural communication groups, neither in the thickness of the flakes (K-W IE-G (p) = 0.1702), nor in the carenate index (K-W IE-G (p) = 0.1402). Therefore, no single patter allowed us to distinguish clearly between the learning groups.

Regarding the cores, both the skilled knapper and the verbal communication learners knapped, in all cases, between 90 and 100% of the core perimeter. In contrast, in both the imitation-emulation and gestural communication groups there were cases in which the core perimeters were lower than these percentages, although cores with between 90 and 100% knapping predominate (11 and 14 cases, respectively). In fact, in the imitation-emulation group four cores had less than 60% of their perimeter knapped.

Although we found no clear differences in core perimeter, the expert knapper exploited the cores more fully, that is, he extracted more mass *per* core, as shown by the mean of the Extracted Mass Indexes obtained for his cores (EMI EK) = 0.76). The verbal communication group produced the EMI most similar to that of the expert knapper (EMI V = 0.71), followed by the gestural communication group (EMI G = 0.63). The least similar EMI was found in the imitation-emulation group, which obtained the lowest mean value (EMI I-E = 0.53), so it is the learning group that extracts less mass *per* core. These differences were statistically significant between the imitation-emulation group and both the verbal communication group (ANOVA I-E-V (p) = 0.01407) and the expert knapper (ANOVA I-E-EK (p) = 0.001407) (Supplementary Material Tables S12 and S13).

The correlation between the cortical surface remaining in the cores and the extracted mass –that is, working out the area of the removals regarding the total surface of the core-, inform about how invasive (marginal or deep) are the flakes relative to the extracted mass. This correlation shows further differences between the skilled knapper and the apprentices, as well as between the different groups of apprentices, depending on the communication conditions. On face A of the cores (the first face of percussion) we found statistically significant differences between all possible pairings, except for the gestural and verbal communication groups (K-W p = 0.8181). In contrast, on face B we found no statistically significant differences between the groups, with the exception of the ratios obtained by the imitation-emulation group and the expert knapper (K-W p = 0.00307). (Supplementary material Tables S14–S17).

When comparing the results from both faces of the cores, the skilled knapper tended to make more invasive removals in the first percussion face (face A, or second face of exploitation). This is established by the mean of the ratio between the cortical surface and the remaining mass on this face, which is much lower than that of the second face of percussion. In the gestural and verbal communication groups this pattern is repeated, although it is less pronounced, whereas in the imitation-emulation group there are very few differences between the first and second percussion faces.

## Discussion

Beside the fact that the timeframe for processing the new knowledge of knapping techniques used in our experiment was relatively short, and considering that transmission of knapping techniques during prehistory would have been daily^53–56^, our results provided relevant insight into the role of language during the early stages of lithic knapping knowledge acquisition.

### Technical capabilities

Interestingly, we found no clear differences between the verbal and gestural communication groups at the technical capability level. Both communication types allowed the apprentice to focus on key gestural aspects of lithic knapping (such as percussion angle, percussion force, and so on). In contrast, the imitation-emulation group struggled to generate successful blows. This difficulty had an impact on the Efficacy Index (EfI), which was higher in the taught groups (gestural and verbal) than for imitation-emulation knappers.

It has been suggested that the acquisition of technical gestures in lithic knapping occurred mainly through demonstration and subsequent reproduction^57,58^, without the need for complex language. However, our results agree with contrasting studies, such as Morgan *et al*., with regard to variables referring to technical capabilities. This is the case, for example, for the number of *“viable flakes per minute”* and the *“probability of a viable flake per hit”*, where gestural and verbal communication learners achieved better results than their counterparts who learned by means of reverse engineering, imitation and basic teaching^39^. However, in the study by Morgan *et al*., the authors did not compare the apprentices’ results with those of an expert knapper, so there is no reliable yardstick against which the technical skill of the apprentices can be measured.

Regardless of the type of social transmission, in this work we show that trainees never reached the level of results obtained by the expert knapper in terms of: 1) the number of hits performed to reduce the core; 2) the low percentage of percussions without extraction compared to the total number of percussions; 3) the relative weight of percussions with successful extractions; and finally, 4) the Efficacy Index. Although some authors argue that it takes only few minutes to control gestures such as the percussion angle^56,59^, the gestural precision of novice knappers is less effective due to their lack of knapping experience. More practice time would therefore be required in order to correctly learn how to master those gestures, as pointed out by various authors^1,31,44,60–64^. In fact, totally inexperienced knappers are even unable to strike the blank at the right angle to obtain conchoidal fracture^65^.

### Sequences

The differences in the reduction strategies observed among the three groups of apprentices revealed that learning through communication (both verbal and gestural) is more effective strategy for teaching the alternating method than visual observation and subsequent reproduction by an apprentice. The imitation group was only able to produce 13 alternating flakes, meaning that this mechanism of social transmission is not sufficiently effective for the early stages of knapping training.

This relative ineffectiveness of imitation as a mechanism for social transmission is consistent with the results published by Morgan *et al*., in which apprentices had to produce Oldowan flakes^39^. These differences were not recorded in other experiments carried out that did not include a non-teaching learning condition^34,38^, since only differences between verbal communication and gestural communication were taken into consideration^39,40^.

Although knowledge of lithic technology can be transmitted in a non-verbal way^34,38,66–70^, some theoretical analyses have established the fact that high-fidelity mechanisms of information transmission are necessary for the existence of cumulative culture^71,72^. In this regard, the results of our study and other research^39^ indicate that teaching, especially by means of verbal communication, is the most effective social transmission mechanism for learning lithic knapping, and is especially helpful for acquiring and retaining concepts such as core rotation and turning, using negatives of previous blows as percussion platforms for subsequent extractions. In contrast, the gestural communication group were unable to perform the alternating method. As Khreisheh points out, the results of apprentices replicating handaxes and the Levallois method improve considerably with only minimal verbal information on the basics of lithic knapping^62^.

### Products

The imitation-emulation volunteers produced flakes with thinner platforms, while the flake platforms of the verbal and gestural communication groups were thicker, more like to those of the skilled knapper. This may indicate that more adequate percussion platforms were selected by these groups compared with the imitation-emulation knappers^73^.

However, it is more difficult to determine whether the differences in the products obtained by the expert knapper and the apprentices are due to the success (or lack of success) of the technical gestures (*savoir-faire*), or to the application (or not) of the alternating method (*connaissance*), particularly with regard to the imitation group, which produced 96.3% non-alternating flakes.

This is because the alternating method generates longer flakes than those obtained through other knapping strategies, such as the alternate method^41^. Furthermore, some authors claim that the application of the alternating method generates more invasive flakes, that is to say, flakes that penetrate more deeply towards the inside of the blank, allowing for the extraction of more cortical surface^42,49^. This could be why the gestural and verbal communication groups exploited the blanks to a greater degree and more effectively, than the apprentices in the imitation-emulation group. In addition, the communication groups presented lower ratios of cortical area/mass surface, because they produced more invasive flakes. Therefore, the gestural and verbal communication apprentices managed the blanks more similarly to the skilled knapper, than the imitation-emulation apprentices.

The differences between the expert knapper’s results and apprentices’ results when extracting flakes mostly by means of the alternating method (such as in the verbal group) seem to be due to technical gestures and not the actual application of the method. However, as the level of *connaissance* and *savoir-faire* of a knapper can affect the products obtained, this makes it difficult to infer their knowledge of the reduction methods and technical skills in the experimental record^62^. This is also applicable to the skills of prehistoric knappers studied through the archaeological record.

All three groups started with identical blanks, and we found no clear differences in the general morphology of the final products (flakes). On the other hand, the most evident differences were seen during the knapping process: in technical abilities (reflected in the number of percussions and extractions made), and in the knapping sequences. This underscores the need for more information about reduction processes, in order to determine the core reduction strategies used, and hence identify a knapper’s degree of experience from the archaeological record.

### Relationship between lithic technology and language

We are currently far from knowing when, how and why language may have emerged, but there are several hypotheses about its appearance: from a progressive evolution that began two million years ago^39,74^, to a recent occurrence around 100–50ka^75^. However, our results lend weight to the pedagogical hypothesis of language^6^, according to which lithic knapping and language may have co-evolved^5,39,76^. In this way, communicative behaviour oriented at social learning and cumulative culture (in lithic knapping) could have co-evolved together with the first hominins.

Initially, this communicative behaviour would not necessarily have been verbal language^77,78^. As Morgan *et al*., suggest the relative simplicity and duration of the knapping processes from the early technocomplexes, such as the Oldowan, may indicate learning by direct observation. This would have allowed limited transmission among individuals maintaining long-term duration contacts, but would restrict the emergence of innovations and the dissemination speed^39^ of more organised methods of core reduction. However, hominin dependence on lithic technology would have generated a selective process favourable to the development of a more complex social transmission mechanism, allowing more effective diffusion of technical knowledge^24,39^.

Language, the fastest and most efficient way to transmit information, could have lent hominins a great competitive advantage^79^ by favouring the social transmission of knowledge related to lithic technology^39,40^. Nevertheless, lithic technology is not the only area in which more effective social transmission would have been advantageous. For example, language would have been beneficial for the acquisition of other key knowledge in terms of subsistence, including hunting, scavenging, food processing, control of raw materials and water supplies. It would also have been beneficial for developing skills requiring coordinated actions by multiple individuals, such as collective defence^80^.

Likewise, the co-evolutionary dynamics between lithic technology and the development of social behaviour favourable to teaching (and possibly verbal language) could explain the sporadic presence of the alternating method in the early archaeological record (Kanjera South in Kenya, dated as *c*. 2 Ma^50^, and Gadeb, in Ethiopia, dated between 1.4 and 0.7 Ma^51^), which may not have become widespread due to limited social transmission. However, identifying the alternating method in the archaeological record is difficult without the presence of refits. In addition, very few studies have focused on identifying significant attributes of this method in flakes^41^.

Finally, the method for separately analysing the learners’ technical abilities to produce flakes (*savoir-faire*) and their theoretical knowledge of the alternating method (*connaissance*) allowed us to observe that both gestural and verbal communication have greater impact on the transmission of the knapping method than on improving flake production efficiency. Through our work we have confirmed that the combination of individual practice and social learning (whether through direct observation or teaching) is fundamental for learning how to knap. This is the model that Whiten called the “*helical curriculum*”, a sort of interaction according to which, as the learner performs more explorations (individual learning), they will perceive further relevant aspects of the knapping processes carried out by other knappers (social learning) than at the beginning of the learning process^40^. Even so, the use of communication, whether gestural or verbal, in the early stages of learning makes the transmission of information more effective than direct observation.

## Conclusions

This study has five major conclusions. Firstly, regardless of the type of social transmission, in a single training session none of the learners achieved the results obtained by the expert knapper in terms of: (a) the number of blows struck during the core reduction; (b) the percentage of percussions without extraction compared to the total percussions; (c) the relative weight of percussions with successful extractions; and (d) the Efficacy Index. Secondly, volunteers who had to replicate lithic knapping through simple observation and replication (imitation-emulation group) had difficulties making successful extractions. This is reflected in the Efficacy Index, which is lower in the imitation-emulation group than in the taught groups (gestural and verbal). Thirdly, the gestural and verbal communication groups selected their percussion platforms in much the same way that the skilled knapper did, whereas the imitation-emulation group did not. Fourthly, the fact that the imitation-emulation group was almost unable to replicate the alternating method leads us to believe that imitation is not sufficient to learn this method, at least in the initial stages of lithic knapping apprenticeship. Lastly, although no significant differences were found between the gestural and verbal communication learners with regard to their ability to replicate the alternating method while knapping under the tuition of the skilled knapper, *the two groups diverged with regard to their ability to retain the information needed to replicate the method on their own*. Only the verbal communication learners were able to knap the core using the alternating method in Phase 2 of the experiment, when they were untutored.

All this leads us to confirm, through the quantification of actions, that gestural and verbal communication allows knappers to acquire knowledge more effectively and efficiently compared to other mechanisms of social transmission, such as simple observation. It is more effective because much more information is transmitted; and more efficient because it achieves results in less time. This effectiveness lies in the fact that both mechanisms (gestural and verbal) facilitate learning by centring the learner’s attention both on the gestural aspects of lithic knapping (i.e., flaking paths, percussion angles, and so on), and theoretical aspects (i.e., understanding and applying the alternating method). In our case, these two mechanisms meant the apprentices focused on the more relevant aspects of the alternating method, such as the use of previous extractions as subsequent percussion platforms, and the production of consecutive sequences of extractions along the perimeter of the core.

Beyond this, verbal communication, unlike gestural communication, allows for the use of concepts such as “percussion platform”, which facilitates the understanding and retention of this knowledge, thereby favouring its subsequent use by the apprentices. The result is that verbal language is more effective and efficient than gestural language for acquiring and retaining key information relating to the technological aspects of knapping.

In summary, in the early stages of apprenticeship, learning by imitation-emulation results in a limited success in flake production through a simple but systematic method, such as alternating knapping, and a little of the scarce knowledge acquired is retained, as the pronounced drop in learned information between Phase 1 and Phase 2 (about 63%) shows. Learning by gestures is a successful method for replicating the alternating method under the tuition of a master knapper, but it proves to be a somewhat inefficient mechanism of social transmission when it comes to retaining the information received (c. 9% of success in Phase 2 compared with Phase 1). Finally, verbal communication plays a major role in the retention of knowledge on lithic knapping and the successful achievement of the alternating method, since the apprentice knappers enjoyed more than a 66% success rate in Phase 2 compared to Phase 1.

Therefore, in a society whose subsistence was highly dependent on lithic technology, natural selection may likely have favoured increasingly complex communicative behaviour, which, in terms of lithic technology, would have improved the efficiency of knowledge transmission. In the same way, the increased complexity of the lithic technology would in turn have involved the development of more complex communicative behaviour capable of transmitting yet more information in a shorter time frame.

Finally, in order to infer the role of verbal language in learning processes on a larger time scale, long-term experiments must be conducted to determine whether or not these differences found in the initial learning phase are maintained, reduced or increased as the knapping experience of the learners increases.

## Materials and Methods

### Experimental design

As described above, this experimental programme involved the participation of 30 volunteers, aged between 20 and 42. These were randomly divided into three groups of 10 individuals each, to study the knapping techniques through imitation-emulation, gestural-communication, and verbal-communication. Participants were asked to reproduce what the expert knapper was doing, i.e., the alternating method. None of them had prior experience of lithic knapping.

Each participant had a single learning session with the same expert knapper, M. Guardiola (Phase 1), with a duration between five and fifteen minutes, depending on the time the novices needed to reduce the blank, as well as a further evaluation session (Phase 2) thirty minutes later, with a duration between five and ten minutes, in which apprentices had to demonstrate what they had learned in the previous phase, without the presence of the skilled knapper. Each session was recorded using a Sony HDR-XR200 video camera. In Phase 1, the imitation-emulation group had to imitate or try to emulate the skilled knapper during his knapping process, without any kind of interaction between the expert and the apprentice. Participants in the group learning through gestural communication were allowed to interact with the teacher only by means of gestures. Finally, participants of the verbal communication group had the benefit of both observing the expert knapping and receiving verbal instructions from him, as well as could ask questions about the process.

Each participant was briefed on the experimental procedure and their consent for the participation in the study and the publication of images of the procedure was required to proceed. All the experiments were performed following the institutional guidelines and regulations for ethical approval (IPHES-URV sim. PGPRL-04-01).

**Figure 6:**
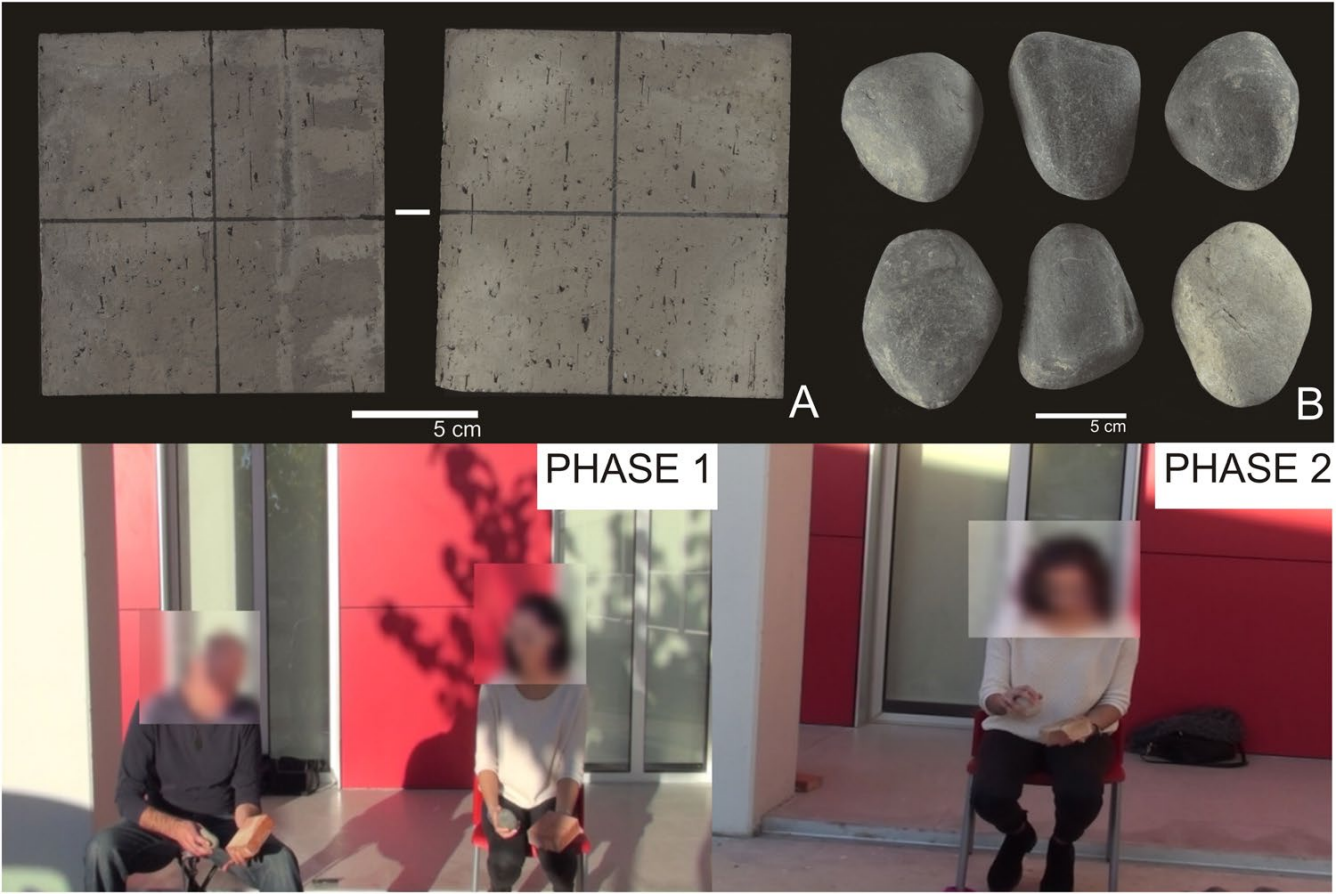
(**A**) Brick used as a blank, (**B**) set of hammerstones used in the experiment. Phase 1: expert knapper and an apprentice knapping; Phase 2: apprentice knapping alone.

As in previous studies^65^, each volunteer was supplied with two commercial bricks with homogeneous textures, morphologies, volumes, and dimensions (134 × 131 × 44 mm) (Fig. 6), one for the learning phase (Phase 1) and the other for the evaluation phase (Phase 2). We decided to use commercial bricks as raw material because they display conchoidal fracture, allow the variables of the initial blank morphology to be controlled, and are safe for inexperienced knappers. If an accidental fracture occurred, participants were not supplied with a new blank but instead, they had to continue knapping one of the fragments. Therefore, the raw material was identical for each volunteer and all volunteers had the same opportunities. Similarly, were supplied with a set of hammerstones of different materials and morphologies, to select according to their criteria.

### Data acquisition and statistical analysis

In order to address both the acquisition of motor skills and the effectiveness of the transmission methods, with regard to *savoir-faire* and *connaissance*, several analyses were undertaken focusing on three units of information: technical capabilities (reflected by actions); products (cores and flakes); and the sequences of gestures and actions that defined the alternating method. Finally, the results of both the teacher and the different learning groups were analysed, according to the phase of the experiment.

For *technical capabilities*, we focused on the actions made during the reduction process, that reflect the acquisition of technical knapping gestures. We reviewed the video-recorded sessions to analysed the knapping sequences carried out by both the apprentices and the skilled knapper. Within each sequence, a set of basic actions related to flakes removal capability was recorded: Percussion (P) = percussion without removal; Erring percussion (D) = percussion resulting in knapping errors (reflected flakes, accidental core fractures, and so on); Extraction of flake (E) = percussion with flake removal; and Failure (F) = percussion action that did not hit the core. As well as counting the actions, this catalogue allowed us to calculate the Efficacy Index (EfI), which calculated by dividing the mass extracted from each blank by the number of times that each participant struck the blank during the experiment. This revealed the relationship between the mass extracted by each volunteer and the number of percussions performed, indicating the effectiveness of each knapper.

We used correspondence analyses to assess the relationships, similitudes and differences in the technical capabilities of the apprentices working under the different learning conditions during the experiment.

The *study of the products* (cores and flakes) was conducted separately. For the flakes, we analysed the technical dimensions (length, width and thickness), the elongation index (the technical length divided by the technical width, according to Bordes^81^), carenate index (smallest dimension (length or width) divided by the thickness, according to Bordes^81^ and Laplace^82^) and the characteristics of the striking platform (platform width and thickness, and cortical area). Because these are all non-parametric variables (Saphiro-Wilk test (p) < 0.05; see Supplementary Material Table S2), we checked the differences observed between the groups for each variable by applying the Kruskal-Wallis (K-W) test, which allowed us to compare the variance in the medians of non-parametric univariate groups. Similarly, we compared the value distribution using the Kolmogorov-Smirnov (K-S) test. This method consists of comparing the distribution of the values of two independent groups, assuming the null hypothesis according to which the original populations have an equal distribution. All statistical analyses were performed using Past v.2.17c software.

For the cores, we studied three parameters: 1) the percentage of the perimeter knapped; 2) the Extracted Mass Index (EMI); and 3) the cortical surface/mass ratio. To obtain the EMI we weighed each core after the experiment had finished, obtaining its mass after the knapping process. We excluded broken cores from the analysis. This value was subtracted from the original mass, to determine the mass extracted during the reduction process. Next, we selected the core with the highest value and used it as a model, dividing the mass extracted from each core by this maximum value. Thus, we obtained a value on a scale of 0 to 1 for each core. The value 1 corresponded to the core used as a reference, i.e., the one from which the greatest quantity of mass was extracted, while the value 0 corresponded to an initial blank, with no removal. The value of this index for each core allowed us to make direct comparisons of the mass extraction for the whole sample.

Because it is a parametric variable (Saphiro-Wilk test (p) > 0.05; see Supplementary Material Table S2), we checked the differences observed between each group for each variable by applying the ANOVA test, which allowed us to compare the variance in the means of the parametric univariate groups.

To obtain the cortical surface/mass ratio we calculated the area of the cortical surface remaining on the two faces of the cores, using the ImageJ v.1.8.0_112 software, taking into account the fact that the cortical surface in bricks is completely flat. To do this, we first established the number of pixels corresponding to one centimetre of the image, using the scale of each photograph. Next, the cortical surface was delimited by taking referential points all around the entire perimeter. Because the cores of some participants accidentally broke during knapping, we established an index consisting of the area obtained from the cortical surface on each face divided by the weight of the knapped core. This converted absolute measurements into ratios, allowing us to compare and quantify the relationship between the decorticated area and a core’s loss of mass.

The *analysis of sequences*, is based on the premise that for a knapping sequence to be alternating, there must be extraction on face A (the first percussion face) followed by a turn of the core, and a subsequent extraction on face B (the second percussion face), taking advantage of the first extraction. Then, the latest removal on face B must be used as a percussion platform for the next extraction on face A, and so on. In addition, for a sequence to be completely alternating there must be continuous rotation along the perimeter of the blank.

Starting from this premise, the three elements of the flakes once refitted allowed us to objectively identify whether or not an alternating sequence had been achieved. Firstly, the percussion face of the core retained on the striking platform of the flake showed whether the knapper alternated between the core faces. Secondly, the cortical surface of the striking platform allowed us to observe if the negative of the previous removal had been used as the percussion platform for the subsequent detachment. Thirdly, the consecutive order of the flakes showed whether the rotation and exploitation around the perimeter of the core was continuous throughout the sequence. These three elements objectively determined whether the alternating method had been properly implemented.

### ETHICS

The authors declare that each participant was briefed on the experimental procedure and their consent for the participation in the study and the publication of images of the procedure was required to proceed. All the experiments were performed following the institutional guidelines and regulations for ethical approval (IPHES-URV sim. PGPRL-04-01).

## Electronic supplementary material

Supplementary material
