# Supplementary material for: Teaching to make stone tools: new experimental evidence supporting a technological hypothesis for the origins of language
Source: Sci Rep. 2017 Oct 31;7:14394. doi: 10.1038/s41598-017-14322-y (PMC5663762; doi:10.1038/s41598-017-14322-y)
Supplement: Supplementary file 1 — Supplementary material [file 41598_2017_14322_MOESM1_ESM.pdf]

Teaching to make stone tools: new experimental evidence supporting a technological hypothesis for the origins of language.

D. Lombao, M. Guardiola & M. Mosquera

Participant	Gender	Age
I-E 1	Male	24
I-E 2	Female	32
I-E 3	Female	24
I-E 4	Female	24
I-E 5	Female	25
I-E 6	Male	26
I-E 7	Female	26
I-E 8	Male	27
I-E 9	Male	25
I-E 10	Male	25
G 1	Female	22
G 2	Female	25
G 3	Male	23
G 4	Male	24
G 5	Male	24
G 6	Male	25
G 7	Female	22
G 8	Female	23
G 9	Female	23
G 10	Female	22
V 1	Female	20
V 2	Female	20
V 3	Male	24
V 4	Male	23
V 5	Male	25
V 6	Female	25
V 7	Female	42
V 8	Female	31
V 9	Male	25
V 10	Female	23
EK	Male	45

Supplementary Table S1. List of participants of the experiment. I-E: imitation-emulation group; G: gestural communication group; V: verbal communication group; EK: expert knapper.

Saphiro-Wilk W (p)	Imitation-Emulation	Gestural communication	Verbal communication	Expert knapper
**Technical capacities**				
P *(n)*	0.1154	0.0004819	0.0549	0.07583
D *(n)*	0.05001	5.765E-06	0.0185	0.1426
E *(n)*	0.5931	0.002536	0.09783	0.2228
F *(n)*	4.984 E-05	6.461 E-05	2.781 E-05	-
Total actions *(n)*	0.1219	0.0001078	0.0766	0.523
Efficacy Index (EfI)	0.0124	0.005934	0.01757	0.004915

Flakes				
Length	4.196 E-07	1.859 E-08	8.553 E-05	8.224 E-09
Weight	6.473 E-07	0.0003741	0.003396	1.925 E-06
Thickness	6.608 E-13	5.194 E-12	4.461 E-09	2.158 E-08
Elongation Index	1.221 E-13	5.07 E-14	6.988 E-16	2.054 E-19
Carenate Index	8.505 E-10	1.905 E-11	4.619 E-16	2.06 E-14
Weight platform	8.356 E-07	2.544 E-06	0.008305	2.014 E-07
Thickness platform	2.841 E-08	4.06 E-12	0.0001083	3.048 E-09
Cores				
Extracted Mass Index (EMI)	0.9132	0.9904	0.3898	0.93
Ratio Cortical Surface (A)/ Extracted mass	0.01726	0.1416	9.012 E-06	3.356 E-09
Ratio Cortical Surface (B)/ Extracted mass	0.9474	0.07076	0.004254	0.4205

Supplementary Table S2. Results of Saphiro-Wilk test of normality for all the variables. P: percussion without extraction; D: percussion resulting in knapping error; E: percussion with flake removal; F: failure.

Participant	Phase	Percussion without extraction (P)	Percussion resulting in knapping error (D)	Percussion with flake removal (E)	Failure (F)	Total
I-E 1	1	40	11	7	0	58
	2	150	14	8	0	172
I-E 2	1	19	8	18	0	45
	2	16	5	16	0	37
I-E 3	1	55	10	24	2	91
	2	68	18	26	3	115
I-E 4	1	65	7	5	0	77
	2	168	18	16	0	202
I-E 5	1	86	7	2	0	95
	2	258	10	18	0	286
I-E 6	1	163	8	25	1	197
	2	82	11	35	1	129
I-E 7	1	111	4	1	0	116
	2	175	11	13	0	199
I-E 8	1	185	5	10	3	203
	2	354	6	16	1	377
I-E 9	1	50	10	17	0	77
	2	89	10	6	0	105
I-E 10	1	172	4	16	4	196
	2	232	4	25	3	264
G 1	1	32	7	16	1	56
	2	34	6	18	6	64
G 2	1	138	11	9	2	160
	2	88	5	21	0	114
G 3	1	26	5	16	0	47
	2	38	14	11	0	63
G 4	1	85	5	20	4	114
	2	103	15	15	7	140
G 5	1	43	8	17	5	73

	2	219	41	35	3	298
G 6	1	41	5	17	0	63
	2	28	4	14	1	47
G 7	1	61	10	12	0	83
	2	30	9	18	1	58
G 8	1	51	6	13	0	70
	2	12	0	11	0	23
G 9	1	46	9	19	0	74
	2	18	4	16	0	38
G 10	1	61	6	17	0	84
	2	62	4	9	0	75
V 1	1	77	9	16	0	102
	2	60	4	13	4	81
V 2	1	77	4	16	1	98
	2	126	9	11	2	148
V 3	1	71	13	23	5	112
	2	49	16	15	1	81
V 4	1	16	6	18	1	41
	2	14	4	10	0	28
V 5	1	48	7	12	1	68
	2	34	4	10	0	48
V 6	1	53	1	17	0	71
	2	75	12	25	0	112
V 7	1	37	4	17	0	58
	2	37	3	12	0	52
V 8	1	25	13	14	0	52
	2	27	5	12	2	46
V 9	1	22	5	18	0	45
	2	9	3	15	0	27
V 10	1	31	6	16	0	53
	2	24	5	16	1	46
EK (I-E 1)	1	24	6	21	0	51
EK (I-E 2)	1	3	8	18	0	29
EK (I-E 3)	1	7	5	18	0	30
EK (I-E 4)	1	21	3	22	0	46
EK (I-E 5)	1	7	1	19	0	27
EK (I-E 6)	1	6	6	21	0	33
EK (I-E 7)	1	15	5	21	0	41
EK. (I-E 8)	1	12	5	26	0	43
EK (I-E 9)	1	21	0	25	0	46
EK (I-E 10)	1	22	3	27	0	52
EK (G 1)	1	2	4	20	0	26
EK (G 2)	1	7	3	11	0	21
EK (G 3)	1	4	1	14	0	19
EK (G 4)	1	8	2	24	0	34
EK (G 5)	1	12	0	24	0	36
EK (G 6)	1	7	2	20	0	29
EK (G 7)	1	10	2	21	0	33
EK (G 8)	1	18	2	15	0	35
EK (G 9)	1	9	1	12	0	22
EK (G 10)	1	13	7	21	0	41
EK (V 1)	1	13	2	16	0	31
EK (V 2)	1	9	6	19	0	34

EK (V 3)	1	10	2	24	0	36
EK (V 4)	1	2	5	19	0	26
EK (V 5)	1	11	6	19	0	36
EK. (V 6)	1	5	4	20	0	29
EK (V 7)	1	14	2	25	0	41
EK. (V 8)	1	13	0	21	0	34
EK (V 9)	1	6	3	24	0	33
EK. (V 10)	1	4	3	23	0	30

Supplementary Table S3. Number and type of actions done by each participant. I-E: imitation-emulation group; G: gestural communication group; V: verbal communication group; EK (I-E): expert knapper when knapping with the imitation-emulation group participants; EK (G): expert knapper when knapping with the gestural group participants; EK (V): expert knapper when knapping with the verbal group participants.

**Kruskal-Wallis Test (p) (K-W)**	**P** (*n*) **Imitation-emulation**	**P** (*n*) **Gestural communication**	**P** (*n*) **Verbal communication**	**P** (*n*) **Expert Knapper**
**P** (*n*) **Imitation-emulation**	0	0.005113	0.0004831	8.63E-09
**P** (*n*) **Gestural com.**	-	0	0.2914	1.828E-08
**P** (*n*) **Verbal com.**	-	-	0	8.77E-06
**P** (*n*) **Expert knapper**	-	-	-	0
**Kolmogorov-Smirnov Test (p) (K-S)**	**P** (*n*) **Imitation-emulation**	**P** (*n*) **Gestural communication**	**P** (*n*) **Verbal communication**	**P** (*n*) **Expert Knapper**
**P** (*n*) **Imitation-emulation**	0	0.0112	0.0007253	1.266E-09
**P** (*n*) **Gestural com.**	-	0	0.6276	1.266 E-09
**P** (*n*) **Verbal com.**	-	-	0	2.151E-07
**P** (*n*) **Expert knapper**	-	-	-	0
**Kruskal-Wallis Test (p) (K-W)**	**D** (*n*) **Imitation-emulation**	**D** (*n*) **Gestural communication**	**D** (*n*) **Verbal communication**	**D** (*n*) **Expert Knapper**
**D** (*n*) **Imitation-emulation**	0	0.2366	0.04693	1.412E-09
**D** (*n*) **Gestural com.**	-	0	0.3064	0.0001305
**D** (*n*) **Verbal com.**	-	-	0	0.001809
**D** (*n*) **Expert knapper**	-	-	-	0
**Kolmogorov-Smirnov Test (p) (K-S)**	**D** (*n*) **Imitation-emulation**	**D** (*n*) **Gestural communication**	**D** (*n*) **Verbal communication**	**D** (*n*) **Expert Knapper**
**D** (*n*) **Imitation-emulation**	0	0.4973	0.1349	5.571 E-05
**D** (*n*) **Gestural com.**	-	0	0.771	0.0007341
**D** (*n*) **Verbal com.**	-	-	0	0.01003
**D** (*n*) **Expert knapper**	-	-	-	0
**Kruskal-Wallis Test (p) (K-W)**	**E** (*n*) **Imitation-emulation**	**E** (*n*) **Gestural communication**	**E** (*n*) **Verbal communication**	**E** (*n*) **Expert Knapper**
**E** (*n*) **Imitation-emulation**	0	0.664	0.9566	0.01236
**E** (*n*) **Gestural com.**	-	0	0.568	0.0003494
**E** (*n*) **Verbal com.**	-	-	0	0.0001124
**E** (*n*) **Expert knapper**	-	-	-	0

Kolmogorov-Smirnov Test (p) (K-S)	E (*n*) Imitation-emulation	E (*n*) Gestural communication	E (*n*) Verbal communication	E (*n*) Expert Knapper
E (*n*) Imitation-emulation	0	0.2219	0.2753	0.00186
E (*n*) Gestural com.	-	0	0.9655	0.0002734
E (*n*) Verbal com.	-	-	0	1.794 E-05
E (*n*) Expert knapper	-	-	-	0
**Kruskal-Wallis Test (p) (K-W)**	**F (*n*) Imitation-emulation**	**F (*n*) Gestural communication**	**F (*n*) Verbal communication**	**F (*n*) Expert Knapper**
F (*n*) Imitation-emulation	0	0.5772	0.9158	0.0002078
F (*n*) Gestural com.	-	0	0.6877	6.981 E-05
F (*n*) Verbal com.	-	-	0	6.875 E-05
F(*n*) Expert knapper	-	-	-	0
**Kolmogorov-Smirnov Test (p) (K-S)**	**F (*n*) Imitation-emulation**	**F (*n*) Gestural communication**	**F (*n*) Verbal communication**	**F (*n*) Expert Knapper**
F (*n*) Imitation-emulation	0	0.9655	0.9999	0.03048
F (*n*) Gestural com.	-	0	0.9655	0.01003
F (*n*) Verbal com.	-	-	0	0.01003
F(*n*) Expert knapper	-	-	-	0
**Kruskal-Wallis Test (p) (K-W)**	**Total (*n*) Imitation-emulation**	**Total (*n*) Gestural communication**	**Total (*n*) Verbal communication**	**Total (*n*) Expert Knapper**
Total (*n*) Imitation-emulation	0	0.005784	0.000684	1.222E-08
Total (*n*) Gestural com.	-	0	0.2233	2.445 E-07
Total (*n*) Verbal com.	-	-	0	4.446 E-06
Total (*n*) Expert knapper	-	-	-	0
**Kolmogorov-Smirnov Test (p) (K-S)**	**Total (*n*) Imitation-emulation**	**Total (*n*) Gestural communication**	**Total (*n*) Verbal communication**	**Total (*n*) Expert Knapper**
Total (*n*) Imitation-emulation	0	0.008162	0.002571	1.266 E-09
Total (*n*) Gestural com.	-	0	0.2753	2.598 E-08
Total (*n*) Verbal com.	-	-	0	2.94 E-06
Total (*n*) Expert knapper	-	-	-	0

Supplementary Table S4. Results of Kruskal-Wallis test (K-W) and Kolmogorov-Smirnov test (K-S). P: percussion without extraction; D: percussion resulting in knapping error; E: percussion with flake removal; F: failure; Total: total number of actions.

Participant	Phase	Efl I-E	Efl G	Efl V	Efl EK (I-E)	Efl EK (G)	Efl EK (V)
1	1	11.8845	8.8661	11.7284	14.7451	25.9577	32.2742
	2	2.8552	16.3328	12.5654			
2	1	15.7267	2.6219	6.4306	23.1379	40.9143	21.8794
	2	18.2432	3.5439	2.5791			
3	1	5.8077	19.6426	5.4107	23.7933	40.8789	18.9250
	2	5.3609	17.2333	8.2938			
4	1	12.5857	6.2605	25.8707	14.3783	17.5412	29.0462
	2	2.8668	2.5679	32.6214			
5	1	1.1547	11.7808	14.7765	27.2852	18.8500	18.3222
	2	1.2951	2.2326	15.9979			

6	1	4.5629	9.7857	13.9634	32.2788	24.2586	25.2310
	2	5.9264	10.8872	8.5723			
7	1	0.1733	8.2446	11.8948	19.0951	20.7212	15.8829
	2	1.9879	9.8914	14.5385			
8	1	1.1621	11.2729	10.6404	14.6000	25.9114	17.1206
	2	1.2332	34.0739	13.3696			
9	1	11.4740	8.7514	14.6000	15.2826	37.6227	19.0152
	2	8.2305	13.9895	28.2296			
10	1	5.0597	7.7929	12.8755	15.0019	17.1732	24.0733
	2	2.0777	2.9373	14.9630			

Supplementary Table S5. Efficacy Index (EfI) for each participant. I-E: imitation-emulation group; G: gestural communication group; V: verbal communication group; EK (I-E): expert knapper when knapping with the imitation-emulation group participants; EK (G): Expert knapper when knapping with the gestural group participants; EK (V): Expert knapper when knapping with the verbal group participants.

Kruskal Wallis Test (p) (K-W)	EfI (*n*) Imitation-emulation	EfI (*n*) Gestural communication	EfI (*n*) Verbal communication	EfI (*n*) Expert Knapper
**EfI (*n*) Imitation-emulation**	0	0.0239	0.0003382	1.57 E-08
**EfI (*n*) Gestural com.**	-	0	0.07205	1.287 E-06
**EfI (*n*) Verbal com.**	-	-	0	2.463 E-05
**EfI (*n*) Expert knapper**	-	-	-	0

Supplementary Table S6. Results of Kruskal-Wallis test (K-W) test for Efficacy Index (EfI).

Participant	Phase	Alternating flakes (*n*)	Non-Alternating flakes (*n*)	Total flakes (*n*)
I-E 1	1	0	23	23
	2	0	15	15
I-E 2	1	9	14	23
	2	0	20	20
I-E 3	1	0	25	25
	2	4	23	27
I-E 4	1	0	12	12
	2	0	18	18
I-E 5	1	0	6	6
	2	0	25	25
I-E 6	1	0	28	28
	2	0	29	29
I-E 7	1	0	5	5

	2	0	13	13
I-E 8	1	0	9	9
	2	0	16	16
I-E 9	1	0	18	18
	2	0	10	10
I-E 10	1	0	13	13
	2	0	22	22
G 1	1	12	4	16
	2	5	10	15
G 2	1	4	14	18
	2	0	17	17
G 3	1	11	4	15
	2	4	10	14
G 4	1	6	16	22
	2	0	18	18
G 5	1	17	2	19
	2	0	30	30
G 6	1	5	12	17
	2	0	14	14
G 7	1	8	6	14
	2	0	23	23
G 8	1	15	1	16
	2	0	11	11
G 9	1	9	10	19
	2	0	20	20
G 10	1	16	3	19
	2	0	10	10
V 1	1	13	5	18
	2	6	9	15
V 2	1	5	13	18
	2	0	13	13
V 3	1	18	6	24
	2	10	11	21
V 4	1	18	1	19
	2	6	6	12
V 5	1	15	1	16
	2	5	10	15
V 6	1	16	1	17
	2	14	8	22
V 7	1	16	0	16
	2	13	0	13
V 8	1	14	1	15
	2	13	1	14
V 9	1	14	6	20
	2	17	0	17
V 10	1	16	2	18
	2	0	18	18

EK (I-E 1)	1	10	7	17
EK (I-E 2)	1	24	0	24
EK (I-E 3)	1	23	0	23
EK (I-E 4)	1	20	0	20
EK (I-E 5)	1	20	1	21
EK (I-E 6)	1	16	1	17
EK (I-E 7)	1	21	0	21
EK (I-E 8)	1	18	0	18
EK (I-E 9)	1	25	0	25
EK (I-E 10)	1	21	4	25
EK (IG 1)	1	24	0	24
EK (G 2)	1	15	0	15
EK (G 3)	1	10	6	16
EK (G 4)	1	22	4	26
EK (G 5)	1	20	1	21
EK (G 6)	1	17	0	17
EK (G 7)	1	14	4	18
EK (G 8)	1	16	1	17
EK (G 9)	1	13	0	13
EK (G 10)	1	20	1	21
EK (V 1)	1	21	0	21
EK (V 2)	1	20	4	24
EK (V 3)	1	23	3	26
EK (V 4)	1	23	0	23
EK (V 5)	1	20	3	23
EK (V 6)	1	22	0	22
EK (V 7)	1	22	4	26
EK (V 8)	1	23	0	23
EK (V 9)	1	22	1	23
EK (V 10)	1	19	0	19

Supplementary Table S7. Number *(n)* of alternating flakes, non-alternating flakes, and total flakes for each participant. I-E: imitation-emulation group; G: gestural communication group; V: verbal communication group; EK (I-E): expert knapper when knapping with the imitation-emulation group participants; EK (G): expert knapper when knapping with the gestural group participants; EK (V): expert knapper when knapping with the verbal group participants.

**Participant**	**Phase**	**Flakes surface A (*n*)**	**Flakes surface B (*n*)**	**Indet. Flakes (*n*)**	**Total flakes (*n*)**
I-E 1	1	10	11	2	23
	2	8	6	1	15
I-E 2	1	12	10	1	23
	2	9	11	0	20
I-E 3	1	12	9	4	25
	2	12	14	1	27
I-E 4	1	9	1	2	12
	2	5	13	0	18
I-E 5	1	4	1	1	6

	2	14	11	0	25
I-E 6	1	9	19	0	28
	2	21	8	0	29
I-E 7	1	1	3	1	5
	2	12	1	0	13
I-E 8	1	5	4	0	9
	2	14	2	0	16
I-E 9	1	14	3	1	18
	2	9	1	0	10
I-E 10	1	8	4	1	13
	2	11	10	1	22
G 1	1	8	7	1	16
	2	8	5	2	15
G 2	1	9	6	3	18
	2	14	2	1	17
G 3	1	8	7	0	15
	2	5	8	1	14
G 4	1	12	10	0	22
	2	7	11	0	18
G 5	1	10	8	1	19
	2	10	13	7	30
G 6	1	8	7	2	17
	2	8	6	0	14
G 7	1	7	6	1	14
	2	12	10	1	23
G 8	1	8	8	0	16
	2	6	5	0	11
G 9	1	11	8	0	19
	2	12	8	0	20
G 10	1	13	6	0	19
	2	7	3	0	10
V 1	1	10	6	2	18
	2	7	7	1	15
V 2	1	12	6	0	18
	2	5	8	0	13
V 3	1	11	13	0	24
	2	12	9	0	21
V 4	1	7	9	3	19
	2	7	5	0	12
V 5	1	9	6	1	16
	2	7	8	0	15
V 6	1	8	8	1	17
	2	12	10	0	22
V 7	1	7	9	0	16
	2	7	6	0	13
V 8	1	8	7	0	15
	2	7	6	1	14

V 9	1	9	10	1	20
	2	9	8	0	17
V 10	1	10	6	2	18
	2	11	7	1	18
EK (I-E 1)	1	8	9	0	17
EK (I-E 2)	1	14	10	0	24
EK (I-E 3)	1	15	8	0	23
EK (I-E 4)	1	9	11	0	20
EK (I-E 5)	1	11	10	0	21
EK (I-E 6)	1	6	9	2	17
EK (I-E 7)	1	9	11	1	21
EK (I-E 8)	1	10	8	0	18
EK (I-E 9)	1	12	11	2	25
EK (I-E 10)	1	16	9	0	25
EK (IG 1)	1	12	11	1	24
EK (G 2)	1	6	7	2	15
EK (G 3)	1	9	7	0	16
EK (G 4)	1	16	10	0	26
EK (G 5)	1	13	8	0	21
EK (G 6)	1	11	6	0	17
EK (G 7)	1	11	7	0	18
EK (G 8)	1	9	7	1	17
EK (G 9)	1	7	5	1	13
EK (G 10)	1	11	10	0	21
EK (V 1)	1	10	11	0	21
EK (V 2)	1	11	12	1	24
EK (V 3)	1	13	13	0	26
EK (V 4)	1	10	13	0	23
EK (V 5)	1	14	9	0	23
EK (V 6)	1	11	11	0	22
EK (V 7)	1	16	10	0	26
EK (V 8)	1	13	10	0	23
EK (V 9)	1	15	8	0	23
EK (V 10)	1	10	9	0	19

Supplementary Table S8. Number (*n*) of flakes from surface A, flakes from surface B, indeterminate flakes and total flakes for each participant. I-E: imitation-emulation group; G: gestural communication group; V: verbal communication group; EK (I-E): expert knapper when knapping with the imitation-emulation group participants; EK (G): expert knapper when knapping with the gestural group participants; EK (V): expert knapper when knapping with the verbal group participants.

Participant	Phase	Consecutive flakes (*n*)	Non-consecutive flakes (*n*)	First flake (*n*)	Indet. Flakes (*n*)	Total flakes (*n*)
I-E 1	1	18	3	1	1	23
	2	11	3	1	0	15
I-E 2	1	14	8	1	0	23
	2	11	8	1	0	20
I-E 3	1	8	16	1	0	25

	2	20	6	1	0	27
I-E 4	1	6	5	1	0	12
	2	9	8	1	0	18
I-E 5	1	4	1	1	0	6
	2	21	3	1	0	25
I-E 6	1	17	10	1	0	28
	2	15	13	1	0	29
I-E 7	1	1	3	1	0	5
	2	11	1	1	0	13
I-E 8	1	0	8	1	0	9
	2	0	15	1	0	16
I-E 9	1	3	14	1	0	18
	2	2	7	1	0	10
I-E 10	1	7	5	1	0	13
	2	6	15	1	0	22
G 1	1	15	0	1	0	16
	2	8	4	1	2	15
G 2	1	16	1	1	0	18
	2	6	10	1	0	17
G 3	1	12	2	1	0	15
	2	7	5	1	1	14
G 4	1	19	2	1	0	22
	2	11	6	1	0	18
G 5	1	14	3	1	1	19
	2	0	29	1	0	30
G 6	1	13	1	1	2	17
	2	4	9	1	0	14
G 7	1	13	0	1	0	14
	2	21	1	1	0	23
G 8	1	13	2	1	0	16
	2	9	1	1	0	11
G 9	1	16	2	1	0	19
	2	5	14	1	0	20
G 10	1	16	2	1	0	19
	2	5	4	1	0	10
V 1	1	14	3	1	0	18
	2	9	4	1	1	15
V 2	1	17	0	1	0	18
	2	9	3	1	0	13
V 3	1	23	0	1	0	24
	2	16	4	1	0	21
V 4	1	14	1	1	3	19
	2	9	2	1	0	12
V 5	1	15	0	1	0	16
	2	13	1	1	0	15
V 6	1	13	2	1	1	17
	2	14	7	1	0	22

V 7	1	15	0	1	0	16
	2	12	0	1	0	13
V 8	1	14	0	1	0	15
	2	12	0	1	1	14
V 9	1	17	1	1	1	20
	2	16	0	1	0	17
V 10	1	17	0	1	0	18
	2	17	0	1	0	18
EK (I-E 1)	1	14	2	1	0	17
EK (I-E 2)	1	22	1	1	0	24
EK (I-E 3)	1	22	0	1	0	23
EK (I-E 4)	1	19	0	1	0	20
EK (I-E 5)	1	19	1	1	0	21
EK (I-E 6)	1	13	1	1	2	17
EK (I-E 7)	1	19	1	1	0	21
EK (I-E 8)	1	16	2	1	0	18
EK (I-E 9)	1	21	0	1	3	25
EK (I-E 10)	1	21	3	1	0	25
EK (IG 1)	1	23	0	1	0	24
EK (G 2)	1	14	0	1	0	15
EK (G 3)	1	13	2	1	0	16
EK (G 4)	1	24	1	1	0	26
EK (G 5)	1	20	0	1	0	21
EK (G 6)	1	16	0	1	0	17
EK (G 7)	1	16	1	1	0	18
EK (G 8)	1	16	0	1	0	17
EK (G 9)	1	9	2	1	1	13
EK (G 10)	1	18	2	1	0	21
EK (V 1)	1	20	0	1	0	21
EK (V 2)	1	22	0	1	1	24
EK (V 3)	1	25	0	1	0	26
EK (V 4)	1	22	0	1	0	23
EK (V 5)	1	22	0	1	0	23
EK (V 6)	1	20	1	1	0	22
EK (V 7)	1	21	4	1	0	26
EK (V 8)	1	21	1	1	0	23
EK (V 9)	1	22	0	1	0	23
EK (V 10)	1	17	1	1	0	19

Supplementary Table S9. Number *(n)* of consecutive flakes with the previous removal, non-consecutive flakes with the previous removal, first flake of the sequence, indeterminate flakes (indet.) and total flakes for each participant. I-E: imitation-emulation group; G: gestural communication group; V: verbal communication group; EK (I-E): expert knapper when knapping with the imitation-emulation group participants; EK (G): expert knapper when knapping with the gestural group participants; EK (V): expert knapper when knapping with the verbal group participants.

**Participant**	**Phase**	**Nco flakes (*n*)**	**Co flakes (*n*)**	**Nco (Co) flakes (*n*)**	**Co (Nco) flakes (*n*)**	**Indet. flakes(*n*)**	**Total flakes (*n*)**

I-E 1	1	7	9	0	1	6	23
	2	2	7	1	0	5	15
I-E 2	1	10	7	1	1	4	23
	2	2	14	0	1	3	20
I-E 3	1	13	9	1	0	2	25
	2	17	7	0	1	2	27
I-E 4	1	2	8	0	0	2	12
	2	2	16	0	0	0	18
I-E 5	1	0	5	0	0	1	6
	2	0	23	0	1	1	25
I-E 6	1	0	25	0	2	1	28
	2	4	21	0	3	1	29
I-E 7	1	1	1	0	0	3	5
	2	3	9	1	0	0	13
I-E 8	1	4	3	0	1	1	9
	2	2	11	0	1	2	16
I-E 9	1	0	11	0	0	7	18
	2	0	7	0	0	3	10
I-E 10	1	2	5	0	3	3	13
	2	6	9	1	2	4	22
G 1	1	8	2	3	1	2	16
	2	4	6	0	0	5	15
G 2	1	6	5	1	1	5	18
	2	0	15	0	0	2	17
G 3	1	10	2	0	0	3	15
	2	10	3	0	0	1	14
G 4	1	13	7	2	0	0	22
	2	9	7	0	0	2	18
G 5	1	10	4	2	0	3	19
	2	10	17	1	0	2	30
G 6	1	10	5	0	1	1	17
	2	2	12	0	0	0	14
G 7	1	9	4	1	0	0	14
	2	2	18	0	2	1	23
G 8	1	8	4	2	0	2	16
	2	6	3	0	1	1	11
G 9	1	13	6	0	0	0	19
	2	2	14	0	1	3	20
G 10	1	10	4	3	0	2	19
	2	1	7	1	0	1	10
V 1	1	14	2	0	0	2	18
	2	11	3	0	0	1	15
V 2	1	11	6	0	0	1	18
	2	4	8	1	0	0	13
V 3	1	16	4	1	1	2	24
	2	11	4	2	1	3	21
V 4	1	12	1	1	0	5	19

	2	6	2	1	1	2	12
V 5	1	13	2	0	0	1	16
	2	7	5	2	0	1	15
V 6	1	15	1	0	0	1	17
	2	19	2	1	0	0	22
V 7	1	15	1	0	0	0	16
	2	10	2	1	0	0	13
V 8	1	9	1	1	2	2	15
	2	10	1	1	0	2	14
V 9	1	15	1	2	2	0	20
	2	14	1	1	0	1	17
V 10	1	15	2	0	0	1	18
	2	4	13	0	0	1	18
EK (I-E 1)	1	14	3	0	0	0	17
EK (I-E 2)	1	22	1	1	0	0	24
EK (I-E 3)	1	22	1	0	0	0	23
EK (I-E 4)	1	18	1	1	0	0	20
EK (I-E 5)	1	17	3	0	0	1	21
EK (I-E 6)	1	13	1	1	0	2	17
EK (I-E 7)	1	15	3	2	0	1	21
EK (I-E 8)	1	16	1	1	0	0	18
EK (I-E 9)	1	19	2	0	0	4	25
EK (I-E 10)	1	21	4	0	0	0	25
EK (IG 1)	1	22	1	0	0	1	24
EK (G 2)	1	13	1	0	0	1	15
EK (G 3)	1	13	3	0	0	0	16
EK (G 4)	1	19	3	2	0	2	26
EK (G 5)	1	18	2	1	0	0	21
EK (G 6)	1	15	1	1	0	0	17
EK (G 7)	1	15	2	1	0	0	18
EK (G 8)	1	10	3	2	0	2	17
EK (G 9)	1	10	2	0	0	1	13
EK (G 10)	1	17	3	0	1	0	21
EK (V 1)	1	19	1	0	0	1	21
EK (V 2)	1	20	3	0	0	1	24
EK (V 3)	1	22	2	1	0	1	26
EK (V 4)	1	22	1	0	0	0	23
EK (V 5)	1	16	5	1	0	1	23
EK (V 6)	1	19	2	1	0	0	22
EK (V 7)	1	21	2	2	0	1	26
EK (V 8)	1	21	2	0	0	0	23
EK (V 9)	1	20	2	0	1	0	23
EK (V 10)	1	17	1	0	0	1	19

Supplementary Table S10. Number (*n*) and types of percussion platforms: completely non-cortical (Nco); completely cortical (Co); non-cortical dominant [Nco (Co)]; cortical dominant [Co (Nco)]; indet.: cortical area undetermined, and total flakes for each participant. I-E: imitation-emulation group; G: gestural communication group; V: verbal communication group; EK (I-E): expert knapper when knapping with the imitation-emulation group participants; EK (G): expert knapper when knapping with the gestural group

participants; EK (V): expert knapper when knapping with the verbal group participants.

Kruskal-Wallis Test (p) (K-W)	Length I-E	Length G	Length V	Length EK
Length I-E	0	0.4031	0.1412	7.033E-08
Length G	-	0	0.5936	4.364E-05
Length V	-	-	0	0.0004749
Length EK	-	-	-	0
Kolmogorov-Smirnov Test (p) (K-S)	Length I-E	Length G	Length V	Length EK
Length I-E	0	0.3896	0.003771	1.089E-13
Length G	-	0	0.4279	7.21E-06
Length V	-	-	0	0.0005322
Length EK	-	-	-	0
Kruskal-Wallis Test (p) (K-W)	Width I-E	Width G	Width V	Width EK
Width I-E	0	0.34	0.005385	0.002277
Width G	-	0	0.09914	0.01897
Width V	-	-	0	0.7662
Width EK	-	-	-	0
Kolmogorov-Smirnov Test (p) (K-S)	Width I-E	Width G	Width V	Width EK
Width I-E	0	0.5828	0.01823	0.00679
Width G	-	0	0.3151	0.0881
Width V	-	-	0	0.8685
Width EK	-	-	-	0
Kruskal-Wallis Test (p) (K-W)	Thickness I-E	Thickness G	Thickness V	Thickness EK
Thickness I-E	0	0.3251	0.003855	0.0006238
Thickness G	-	0	0.005541	0.001548
Thickness V	-	-	0	0.7548
Thickness EK	-	-	-	0
Kolmogorov-Smirnov Test (p) (K-S)	Thickness I-E	Thickness G	Thickness V	Thickness EK
Thickness I-E	0	0.1702	3.161E-06	2.383E-09
Thickness G	-	0	0.00551	3.753E-05

Thickness V	-	-	0	0.7637
Thickness EK	-	-	-	0
Kruskal-Wallis Test (p) (K-W)	Elong. Index I-E	Elong. Index G	Elong. Index V	Elong. Index EK
Elong. Index I-E	0	0.885	0.0714	0.7823
Elong. Index G	-	0	0.1178	0.6689
Elong. Index V	-	-	0	0.0235
Elong. Index EK	-	-	-	0
Kolmogorov-Smirnov Test (p) (K-S)	Elong. Index I-E	Elong. Index G	Elong. Index V	Elong. Index EK
Elong. Index I-E	0	0.8508	0.1547	0.5649
Elong. Index G	-	0	0.2245	0.8659
Elong. Index V	-	-	0	0.04619
Elong. Index EK	-	-	-	0
Kruskal-Wallis Test (p) (K-W)	Car. Index I-E	Car. Index G	Car. Index V	Car. Index EK
Car. Index I-E	0	0.1042	4.33E-07	5.868E-06
Car. Index G	-	0	0.001969	0.01934
Car. Index V	-	-	0	0.1532
Car. Index EK	-	-	-	0
Kolmogorov-Smirnov Test (p) (K-S)	Car. Index I-E	Car. Index G	Car. Index V	Car. Index EK
Car. Index I-E	0	0.1514	3.161E-06	0.0001759
Car. Index G	-	0	0.001397	0.006241
Car. Index V	-	-	0	0.1095
Car. Index EK	-	-	-	0
Kruskal-Wallis Test (p) (K-W)	Width S. Plat. I-E	Width S. Plat. G	Width S. Plat. V	Width S. Plat. EK
Width S. Plat. I-E	0	0.07126	0.2939	0.179
Width S. Plat. G	-	0	0.4352	0.00137
Width S. Plat. V	-	-	0	0.01352
Width S. Plat. EK	-	-	-	0
Kolmogorov-Smirnov Test (p) (K-S)	Width S. Plat. I-E	Width S. Plat. G	Width S. Plat. V	Width S. Plat. EK
Width S. Plat. I-E	0	0.08092	0.22	0.3987
Width S. Plat. G	-	0	0.8893	0.0007717
Width S. Plat. V	-	-	0	0.003969

Width S. Plat. EK	-	-	-	0
**Kruskal-Wallis Test (p) (K-W)**	**Thickness S. Plat. I-E**	**Thickness S. Plat. G**	**Thickness S. Plat. V**	**Thickness S. Plat. EK**
**Thickness S. Plat. I-E**	0	0.01796	0.001889	0.004595
**Thickness S. Plat. G**	-	0	0.5729	0.9257
**Thickness S. Plat. V**	-	-	0	0.3566
**Thickness S. Plat. EK**	-	-	-	0
**Kolmogorov-Smirnov Test (p) (K-S)**	**Thickness S. Plat. I-E**	**Thickness S. Plat. G**	**Thickness S. Plat. V**	**Thickness S. Plat. EK**
**Thickness S. Plat. I-E**	0	0.05742	0.001898	0.00264
**Thickness S. Plat. G**	-	0	0.4801	0.4915
**Thickness S. Plat. V**	-	-	0	0.0844
**Thickness S. Plat. EK**	-	-	-	0

Supplementary Table S11. Results of Kruskal-Wallis test (K-W) and Kolmogorov-Smirnov test (K-S) for each variable of the flakes. S. Plat.: striking platform.

Participant	Phase	EMI I-E	EMI G	EMI V	EMI EK (I-E)	EMI EK (G)	EMI EK (V)
1	1	-	0.5378	-	0.8145	0.7310	-
	2	0.5319	-	-			
2	1	0.7665	0.4543	0.6826	0.7268	0.9306	0.8057
	2	0.7311	-	0.4134			
3	1	0.5724	1	0.6564	0.7731	0.8413	0.7379
	2	0.6677	-	0.7276			
4	1	-	0.773	-	0.7164	0.6460	0.8180
	2	0.6272	0.3894	0.9893			
5	1	0.1188	-	-	0.7979	0.735	0.7144
	2	0.4012	0.7206	-			
6	1	0.9736	0.7014	-	-	0.762	0.7925
	2	0.8280	0.5542	-			
7	1	0.0217	0.7412	0.7472	-	0.7406	0.7053
	2	0.4285	0.6214	0.8188			
8	1	0.2555	0.8547	0.5993	0.68	-	0.6305
	2	0.5035	-	0.6661			
9	1	-	-	0.7116	0.7614	0.8965	0.6797
	2	-	0.5758	0.8256			
10	1	-	0.709	0.7391	0.8449	0.7626	0.7822
	2	0.5941	0.2386	0.7455			

Supplementary Table S12. Results of Extracted Mass Index (EMI) for each participant. I-E: imitation-emulation group; G: gestural communication group; V: verbal communication group; EK (I-E): expert knapper when knapping with the imitation-emulation group participants; EK (G): expert knapper when knapping with the gestural group participants; EK (V): expert knapper when knapping with the verbal group participants.

ANOVA test (p)	**EMI I-E**	**EMI G**	**EMI V**	**EMI EK**
**EMI I-E**	0	0.3163	0.01407	0.001407
**EMI G**	-	0	0.5105	0.1434
**EMI V**	-	-	0	0.8643
**EMI EK**	-	-	-	0

Supplementary Table S13. Results of ANOVA test for Extracted Mass Index (EMI). I-E: imitation-emulation group; G: gestural communication group; V: verbal communication group; EK: expert knapper.

**Participant**	**Phase**	**Ratio C.S.(A)/E.M. I-E**	**Ratio C.S.(A)/E.M. G**	**Ratio C.S.(A)/E.M. V**	**Ratio C.S.(A)/E.M. EK (I-E)**	**Ratio C.S.(A)/E.M. EK (G)**	**Ratio C.S.(A)/E.M. EK (V)**
1	1	0.0720	0.1174	0.4648	0.0478	0.0615	0.0321
	2	0.1460	0.0813	0.0996			
2	1	0.1544	0.1213	0.0949	0.0277	0.0682	0.1092
	2	0.1420	0.0515	0.1466			
3	1	0.0687	0.0514	0.1334	0.0524	0.0728	0.1250
	2	0.0673	0.0729	0.1454			
4	1	0.2169	0.1262	0.0418	0.578	0.0120	0.0587
	2	0.1390	0.0840	0.0949			
5	1	0.1683	0.1149	0.1503	0	0.0526	0.0734
	2	0.1697	0.0429	0.1197			
6	1	0.1136	0.1120	0.1209	0.0097	0.0034	0.0885
	2	0.1333	0.1429	0.0859			
7	1	0.1656	0.0706	0.0264	0.0452	0.0356	0.0534
	2	0.1559	0.1393	0.0966			
8	1	0.1582	0.0544	0.0453	0.0054	0.0425	0.0262
	2	0.1634	0.1077	0.1070			
9	1	0.1615	0.0873	0.0951	0.0811	0.0691	0.0150
	2	0.1824	0.1428	0.0597			
10	1	0.1560	0.0700	0.0154	0	0.1204	0.0210
	2	0.0632	0.1299	0.0909			

Supplementary Table S14. Results of Ratio Cortical Surface (surface A) divided by the extracted mass for each participant. I-E: imitation-emulation group; G: gestural communication group; V: verbal communication group; EK (I-E): expert knapper when knapping with the imitation-emulation group participants; EK (G): expert knapper when knapping with the gestural group participants; EK (V): expert knapper when knapping with the verbal group participants.

Kruskal-Wallis test (K-W) (p)	**Ratio C.S.(A)/E.M. I-E**	**Ratio C.S.(A)/E.M. G**	**Ratio C.S.(A)/E.M. V**	**Ratio C.S.(A)/E.M. EK**

**Ratio C.S.(A)/E.M. I-E**	0	0.001014	0.004318	1.286 E-06
**Ratio C.S.(A)/E.M. G**	-	0	0.8181	0.0003014
**Ratio C.S.(A)/E.M. V**	-	-	0	0.0009094
**Ratio C.S.(A)/E.M. EK**	-	-	-	0

Supplementary Table S15. Results of Kruskal-Wallis test (K-W) for the Ratio Cortical Surface (surface A) divided by the extracted mass for each participant. I-E: imitation-emulation group; G: gestural communication group; V: verbal communication group; EK: expert knapper.

**Participant**	**Phase**	**Ratio C.S.(B)/E.M. I-E**	**Ratio C.S.(B)/E.M. G**	**Ratio C.S.(B)/E.M. V**	**Ratio C.S.(B)/E.M. EK (I-E)**	**Ratio C.S.(B)/E.M. EK (G)**	**Ratio C.S.(B)/E.M. EK (V)**
1	1	0.0546	0.1245	0.2979	0.1053	0.0426	0.0943
	2	0.1485	0.1763	0.0893			
2	1	0.0747	0.1350	0.1002	0.1059	0.0699	0.0492
	2	0.1248	0.0510	0.1228			
3	1	0.1493	0.0929	0.1052	0.0817	0.0615	0
	2	0.0977	0.0399	0.0998			
4	1	0.0893	0.1358	0.1462	0.0849	0.1655	0.0931
	2	0.1499	0.1121	0.0557			
5	1	0.1638	0.0667	0.0506	0.1592	0.0779	0.1075
	2	0.1588	0.0350	0.1477			
6	1	0.1924	0.0681	0.0603	0	0.1293	0.0532
	2	0.1723	0.1435	0.0763			
7	1	0.1621	0.1481	0.1513	0.1225	0.0998	0.0935
	2	0.1250	0.1614	0.1079			
8	1	0.1390	0.1487	0.1528	0.1235	0.1287	0.1608
	2	0.1222	0.1341	0.1009			
9	1	0.1442	0.0969	0.0682	0.0678	0.1297	0.1144
	2	0.1132	0.1454	0.1039			
10	1	0.1063	0.1293	0.1403	0.1114	0.0684	0.0997
	2	0.1073	0.1616	0.1732			

Supplementary Table S16. Results of Ratio Cortical Surface (surface B) divided by the extracted mass for each participant. I-E: imitation-emulation group; G: gestural communication group; V: verbal communication group; EK (I-E): expert knapper when knapping with the imitation-emulation group participants; EK (G): expert knapper when knapping with the gestural group participants; EK (V): expert knapper when knapping with the verbal group participants.

Kruskal-Wallis test (K-W) (p)	**Ratio C.S.(B)/E.M. I-E**	**Ratio C.S.(B)/E.M. G**	**Ratio C.S.(B)/E.M. V**	**Ratio C.S.(B)/E.M. EK**
**Ratio C.S.(B)/E.M. I-E**	0	0.2977	0.1441	0.00307
**Ratio C.S.(B)/E.M. G**		0	0.7764	0.05992
**Ratio C.S.(B)/E.M. V**			0	0.205
**Ratio**				0

**C.S.(B)/E.M. EK**				

Supplementary Table S17. Results of Kruskal-Wallis test (K-W) for the Ratio Cortical Surface (surface B) divided by the extracted mass for each participant. I-E: imitation-emulation group; G: gestural communication group; V: verbal communication group; EK: expert knapper.
